# Distinct Excitonic Emissions in 2D (C_7_H_7_N_2_)_2_PbX_4_ (X = Cl, Br) under Compression

**DOI:** 10.1002/advs.202305597

**Published:** 2023-11-20

**Authors:** Hai Zhang, Peijie Zhang, Chenlong Xie, Jiang Han, Bin Xu, Zewei Quan

**Affiliations:** ^1^ Department of Chemistry Southern University of Science and Technology (SUSTech) Shenzhen Guangdong 518055 China

**Keywords:** free excitonic emission, high pressure, hybrid metal halides, self‐trapped emission

## Abstract

Two dimensional (2D) hybrid metal halides (HMHs) usually exhibit free excitonic (FE) emission, and self‐trapped excitonic (STE) emission can also be achieved by adopting appropriate halogens and organic cations. Recently, significant efforts have been made to modulate and then clarify the transformation and connection between these two types of excitonic emissions in 2D HMHs. In this study, intriguing pressure‐tuned transitions between FE emission and STE emission are observed in 2D (C_7_H_7_N_2_)_2_PbCl_4_. In contrast, only FE emissions with tunable emission energies are observed in 2D (C_7_H_7_N_2_)_2_PbBr_4_ which possesses a similar structure with (C_7_H_7_N_2_)_2_PbCl_4_ under compression. Such distinct halide‐dependent optical responses under pressure are experimentally revealed to arise from the intricate interplay among several factors in these HMHs, including the stiffness of the structure, the Coulomb force between the organic cations and the inorganic octahedra, and the magnitude of inorganic octahedral distortion. These high‐pressure optical explorations can unravel the underlying interrelationship between the crystal structure and excitonic emission in 2D HMHs.

## Graphical abstract

Two 2D hybrid metal halides, (C_7_H_7_N_2_)_2_PbCl_4_ and (C_7_H_7_N_2_)_2_PbBr_4_, with comparable initial structures exhibit distinct excitonic emissions at ambient conditions and under high pressure due to varied structural stiffness, Coulomb force, and octahedral distortion.

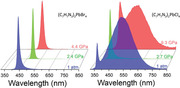

## 1 Introduction

The crystal structure of two‐dimensional (2D) hybrid metal halides (HMH) can be envisioned as “cutting” the three‐dimensional (3D) HMH structure along one crystal plane, forming sheets that incorporate halide ions (Cl, Br, or I) to satisfy the surface metal coordination, with large organic cations inserted between inorganic layers as spacers.^[^ ^1^ ^]^ Usually, typical free excitonic (FE) emission dominates the photoluminescence (PL) in 2D HMH, which is primarily attributed to the inorganic layer and exhibits a small Stokes shift, a narrow full width at half maximum (FWHM), and a short PL lifetime.^[^ ^2^ ^]^ These FE emission characteristics in 2D HMHs provide application prospects in the field of displays.^[^ ^1a^ ^]^ Recently, research attention has also been drawn to self‐trapped excitonic (STE) emissions, which is known for a large Stokes shift and a broad FWHM. Such STE features in 2D HMHs make them also suitable for lighting applications.^[^ ^3^ ^]^ Thus, delicate regulation of excitonic emission modes (FE and STE emissions) in 2D HMHs is of importance for tailoring optical properties for diverse optoelectronic devices. Currently, the modulation of these two excitonic emissions primarily relies on different chemical components, such as halogen type,^[^ ^4^ ^]^ organic cation,^[^ ^5^ ^]^ and metal doping.^[^ ^6^ ^]^

Compared with the traditional chemical methods, high pressure is a distinct physical approach to continuously tune the interatomic distance in materials, without altering their chemical composition. In addition, a series of in situ high‐pressure characterization techniques can simultaneously monitor the variations of crystal structures and optical properties under continuous compression, providing a powerful and reliable approach to investigating novel functional materials.^[^ ^7^ ^]^ As for these HMHs with varied crystal structures, a series of optical variations under high pressure have been demonstrated, including emission generation,^[^ ^8^ ^]^ emission enhancement,^[^ ^9^ ^]^ and emission energy modulation,^[^ ^10^ ^]^ providing novel insight into these intriguing excitonic emissions in HMHs. For example, pressure‐induced emission was demonstrated in 0D (bmpy)_6_[Pb_3_Br_12_] due to the inhibition of phonon‐assisted non‐radiative pathways, persisting up to 80 GPa.^[^ ^11^ ^]^ In addition, the PL quantum yield of 1D C_4_N_2_H_14_PbBr_4_ can reach up to 90% through pressure‐induced suppression of non‐radiative transitions.^[^ ^12^ ^]^ Furthermore, the bandgap of 2D (3AMP)PbI_4_ was obviously reduced under high pressure, resulting in a significant and monotonous redshift of the FE emission, from 561 nm at 1 atm to 735 nm at 7.1 GPa.^[^ ^13^ ^]^ However, the conversion between FE emission and STE emission under high pressure has been rarely explored,^[^ ^14^ ^]^ due to the lack of appropriate HMH systems. This kind of study is essential to uncover the key structural factors in facilitating desired excitonic emissions in HMHs.

In this paper, two 2D Pb‐based HMHs, (C_7_H_7_N_2_)_2_PbBr_4_ and (C_7_H_7_N_2_)_2_PbCl_4_, with similar initial structures have been investigated under high pressure. (C_7_H_7_N_2_)_2_PbBr_4_ consistently exhibits FE emission under ambient conditions and high pressure. In contrast, (C_7_H_7_N_2_)_2_PbCl_4_ initially undergoes a transition from STE emission to FE emission at low pressure, and then generates a new STE emission at a higher pressure. These novel excitonic transitions between STE emission to FE emission in (C_7_H_7_N_2_)_2_PbCl_4_ are systematically investigated based on the corresponding structural variations, enhancing the understanding of the intrinsic structure–property correlation in 2D HMHs.

## 2 Results and Discussion

**Figure 1:**
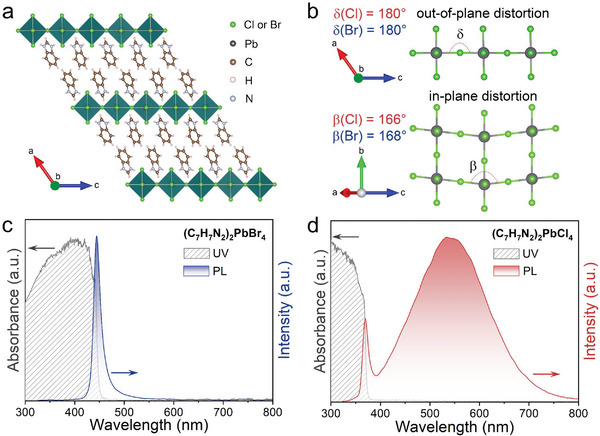
Crystal structures, UV–Vis absorption and PL emission spectra of (C_7_H_7_N_2_)_2_PbBr_4_ and (C_7_H_7_N_2_)_2_PbCl_4_ at ambient conditions. a, b) represent the crystal structures and the detailed views of (C_7_H_7_N_2_)_2_PbBr_4_ and (C_7_H_7_N_2_)_2_PbCl_4_ along the inorganic layer direction. c, d) represent the absorption and PL spectra of (C_7_H_7_N_2_)_2_PbBr_4_ and (C_7_H_7_N_2_)_2_PbCl_4_, respectively.

Single crystals of (C_7_H_7_N_2_)_2_PbCl_4_ and (C_7_H_7_N_2_)_2_PbBr_4_ are synthesized according to the literature.^[^ ^15^ ^]^ The crystal structures of (C_7_H_7_N_2_)_2_PbCl_4_ and (C_7_H_7_N_2_)_2_PbBr_4_ are determined by single‐crystal X‐ray diffraction at room temperature, both of which show a structure with the monoclinic space group C2/c. The powder X‐ray diffraction (PXRD) patterns of these two samples are consistent with their simulated PXRD patterns, confirming the purity of the materials (Figure S1, Supporting Information). In these two compounds, inorganic layers are formed by the corner‐sharing [PbCl_6_]^4−^ and [PbBr_6_]^4−^ octahedra, which are separated by the C_7_H_7_N_2_^+^ cation to form 2D HMHs (**Figure** **1**a). By calculating the octahedral angle distortion $\sigma_{out}^{2}$ and the octahedral bond length distortion *λ* of these two HMHs (see the Supporting Information: Data Analyses),^[^ ^16^ ^]^ it is found that the intra‐octahedral structural distortions are negligible (Table S1, Supporting Information). The inter‐octahedral distortion is divided into in‐plane distortion (*D*_in_) and out‐of‐plane distortion (*D*_out_). The out‐of‐plane distortions (*D*_out_) for both (C_7_H_7_N_2_)_2_PbCl_4_ and (C_7_H_7_N_2_)_2_PbBr_4_ are 180°, while the in‐plane distortions (*D*_in_) are 166° for (C_7_H_7_N_2_)_2_PbCl_4_ and 168° for (C_7_H_7_N_2_)_2_PbBr_4_ (Figure 1b), respectively. Although these two materials have crystal structures and very similar structural distortions, they exhibit significantly different PL spectra. As shown in Figure 1c, (C_7_H_7_N_2_)_2_PbBr_4_ emits a narrow PL emission peak at 444 nm, which is identified as FE emission. On the other hand, (C_7_H_7_N_2_)_2_PbCl_4_ exhibits a strong PL emission peaked at 370 and 534 nm (Figure 1d), which are indexed to FE emission and STE emission, respectively.^[^ ^15^ ^]^ The parameters *k* for FE emission and STE emission of (C_7_H_7_N_2_)_2_PbCl_4_ at ambient conditions are determined to be 1.11 and 1.13 (Figure S2, Supporting Information), respectively, indicating that both emissions are ascribed to exciton recombination^[^ ^17^ ^]^ (the details are discussed in the following section). The absorption edges of (C_7_H_7_N_2_)_2_PbBr_4_ and (C_7_H_7_N_2_)_2_PbCl_4_ are located at 444 and 370 nm (Figure 1c,d), from which their bandgaps are estimated to be 2.77 and 3.34 eV, respectively. It is worth noting that the Huang‐Rhys factors (*S*) for (C_7_H_7_N_2_)_2_PbBr_4_ and (C_7_H_7_N_2_)_2_PbCl_4_ are 13 and 181, respectively.^[^ ^15^ ^]^ Substituting chlorine for bromine in (C_7_H_7_N_2_)_2_PbBr_4_ can influence the thermal vibration between the organic cation and inorganic skeleton, resulting in a tenfold enhancement of electron–phonon coupling. Compared to (C_7_H_7_N_2_)_2_PbBr_4_, (C_7_H_7_N_2_)_2_PbCl_4_ also exhibited lower lattice‐deformation energy and a lower self‐trapping energy, making it more prone to assume a self‐trapped state. Consequently, the PL spectra of (C_7_H_7_N_2_)_2_PbCl_4_ and (C_7_H_7_N_2_)_2_PbBr_4_ differ obviously at ambient conditions.^[^ ^15^ ^]^

**Figure 2:**
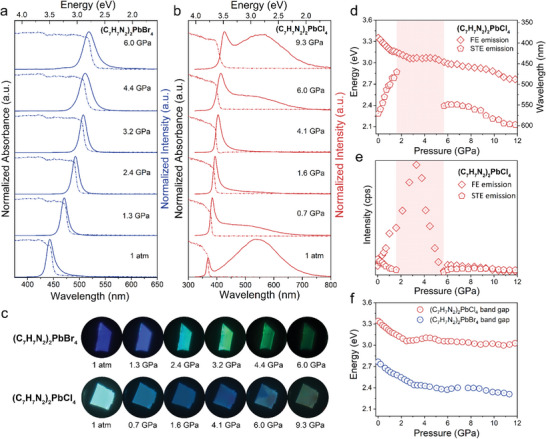
Optical properties of (C_7_H_7_N_2_)_2_PbBr_4_ and (C_7_H_7_N_2_)_2_PbCl_4_ under high pressure. a, b) represent the pressure‐dependent absorption/PL emission spectra of (C_7_H_7_N_2_)_2_PbBr_4_ and (C_7_H_7_N_2_)_2_PbCl_4_, respectively. c) PL micrographs of (C_7_H_7_N_2_)_2_PbBr_4_ and (C_7_H_7_N_2_)_2_PbCl_4_ single crystals under UV‐light irradiation with the increasing pressure. d) The energy evolutions of two PL emission peaks in (C_7_H_7_N_2_)_2_PbCl_4_ with increasing pressure. e) The intensity evolutions of two PL emission peaks in (C_7_H_7_N_2_)_2_PbCl_4_ with the increasing pressure. f) The bandgap evolutions of (C_7_H_7_N_2_)_2_PbBr_4_ and (C_7_H_7_N_2_)_2_PbCl_4_ with the increasing pressure.

To study the variations in optical properties of (C_7_H_7_N_2_)_2_PbBr_4_, in situ PL spectra upon compression to 17.1 GPa are detected under the laser excitation of 355 nm (**Figure** **2**a; Figure S3, Supporting Information). With increasing pressure, the FE emission intensity of (C_7_H_7_N_2_)_2_PbBr_4_ gradually increases and reaches a maximum at 3.5 GPa, accompanied by a redshift in the emission wavelength (Figure 2a; Figure S3a, Supporting Information). With further compression, the FE emission intensity begins to decrease, concomitant with a continuous redshift in emission wavelength. Upon decompression from 17.1 GPa, almost no fluorescence is observed in recovered (C_7_H_7_N_2_)_2_PbBr_4_ (Figure S3c, Supporting Information) at ambient conditions, which is due to pressure‐induced structural collapse. As shown in Figure 2b, the spectral responses of (C_7_H_7_N_2_)_2_PbCl_4_ crystal under 355 nm excitation significantly differ from those of (C_7_H_7_N_2_)_2_PbBr_4_. The complete PL spectra of (C_7_H_7_N_2_)_2_PbCl_4_ under high pressure are shown in Figure S4 (Supporting Information). With increasing pressure to 1.6 GPa, STE emission in (C_7_H_7_N_2_)_2_PbCl_4_ is gradually quenched with a continuous blueshift (Figure 2b,d). Upon further compression to 5.7 GPa, only pure FE emission is observed. In contrast, the FE emission intensity significantly increases and reaches a maximum at 3.3 GPa, followed by a gradual decrease until 5.7 GPa (Figure S4b, Supporting Information). Simultaneously, FE emission exhibits a redshift until reaching 2.5 GPa, followed by slight variations until 5.7 GPa (Figure 2d). After the critical pressure of 6.0 GPa, the PL intensity of (C_7_H_7_N_2_)_2_PbCl_4_ suddenly increases, and a new broadband emission peaked at ≈514 nm begins to appear (Figure 2b; Figure S4c, Supporting Information). With further compression, the broadband emission intensity increases until 8.3 GPa and then decreases, accompanied by a continuous redshift in emission wavelength (Figure 2d,e). Meanwhile, FE‐emission intensity gradually decreases, accompanied by a continuous redshift (Figure 2d,e). Upon decompression from 18.2 GPa, obvious changes are observed in the spectral characteristics of (C_7_H_7_N_2_)_2_PbCl_4_ compared to its initial spectrum (Figure S4d, Supporting Information), which is also ascribed to the presence of structural amorphousness in the recovered (C_7_H_7_N_2_)_2_PbCl_4_.

In order to identify the origin of the new broadband emission in (C_7_H_7_N_2_)_2_PbCl_4_ above 6.0 GPa, the PL lifetimes and the emission intensities of the new peak under varied excitation powers are measured (Figures S6 and S7, Supporting Information). At 8.5 GPa, the average PL lifetimes of FE emission and the new broadband emission under the laser excitation of 375 nm are determined to be 0.62 and 1.37 ns, respectively (Figure S6, Supporting Information). The PL lifetimes are very close, which does not explain their emission characteristics. Thus, the emission intensities of FE emission and the new broadband emission at 7.3 GPa under varied exciton powers are further measured (Figure S7a, Supporting Information). The acquired intensity‐power data are fitted using the power‐law equation of *I = nL^k^* (where *I* represents the intensity of the emission peak and *L* denotes the laser excitation power) to deduce the power exponent of *k*.^[^ ^18^ ^]^ The *k* value has been widely employed to identify the origin of PL emission peaks, where *k* value ranges from 1 to 2 corresponds to exciton recombination, while *k* value less than 1 is associated with impurity/defect‐related recombination.^[^ ^18b^ ^]^ The parameters *k* for FE emission and new broadband emission of (C_7_H_7_N_2_)_2_PbCl_4_ at 7.3 GPa are determined to be 1.11 and 1.07 (Figure S7b, Supporting Information), respectively, indicating that the two emission mechanisms are still associated with exciton recombination.^[^ ^17^ ^]^ Therefore, the new broadband emission is classified as a new STE emission (STE‐2).

In order to trace the evolution of bandgap in (C_7_H_7_N_2_)_2_PbBr_4_ and (C_7_H_7_N_2_)_2_PbCl_4_ under high pressure, UV/Vis absorption experiments are carried out (Figure S8, Supporting Information). With increasing pressure to 6.0 GPa, the bandgap of (C_7_H_7_N_2_)_2_PbBr_4_ continues to decrease (Figure 2f). Above this pressure, the bandgap increases a little bit and then continues to decrease slightly, which could be attributed to a pressure‐induced phase transition. In the case of (C_7_H_7_N_2_)_2_PbCl_4_, as the pressure increases, the absorption edge of (C_7_H_7_N_2_)_2_PbCl_4_ undergoes a continuous redshift until 2.5 GPa, indicating the decrease of bandgap under mild compression (Figure 2f; Figure S8b, Supporting Information). Between 2.5 to 4.5 GPa, the absorption edge of (C_7_H_7_N_2_)_2_PbCl_4_ shows a blueshift with an increased bandgap, indicating possible structural changes in this range (Figure 2f). With further compression, (C_7_H_7_N_2_)_2_PbCl_4_ exhibits a continuous narrowing of the bandgap up to 12.9 GPa. However, upon full decompression from 17–18 GPa, the absorption spectra of (C_7_H_7_N_2_)_2_PbBr_4_ and (C_7_H_7_N_2_)_2_PbCl_4_ cannot be recovered (Figure S8c,d, Supporting Information), which could be attributed to the collapse of the crystal structures under high pressure. It is worth noting that (C_7_H_7_N_2_)_2_PbBr_4_ single crystal can withstand multiple cycles of pressurization up to smaller maximum pressure (≈3 GPa), and the PL and the absorption spectra can recover to their original state upon decompression (Figure S9, Supporting Information). Both the fluorescence peak position and the bandgap energy demonstrate a nearly linear relationship with pressure, with the slope of 19.6 nm GPa^−1^ and −0.098 eV GPa^−1^ (Figure S9c,d, Supporting Information), respectively. Beyond 3.5 GPa, the PL intensity of (C_7_H_7_N_2_)_2_PbBr_4_ decreases significantly and could not be recovered by decompression. According to the following Raman and ADXRD data, this difference is due to the gradual amorphization of (C_7_H_7_N_2_)_2_PbBr_4_ structure under above 3 GPa, resulting in an irreversible transformation of the structure. These intriguing properties indicate their potential application as a tunable pressure sensor.

**Figure 3:**
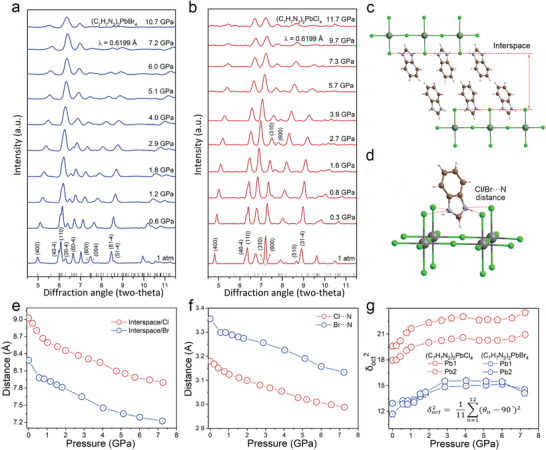
Pressure‐dependent structural evolutions of (C_7_H_7_N_2_)_2_PbBr_4_ and (C_7_H_7_N_2_)_2_PbCl_4_.(a, b) represent the in situ high‐pressure ADXRD patterns of (C_7_H_7_N_2_)_2_PbBr_4_ and (C_7_H_7_N_2_)_2_PbCl_4_, respectively. c) demonstrates the distance between neighboring inorganic layers in these two HMH crystals. d) demonstrates the distance between N atom in organic cation and Cl/Br atom in metal–halide anion. e) High‐pressure evolutions of the distances in (c) in (C_7_H_7_N_2_)_2_PbBr_4_ and (C_7_H_7_N_2_)_2_PbCl_4_, respectively. f) High‐pressure evolutions of the distances in (d) in (C_7_H_7_N_2_)_2_PbBr_4_ and (C_7_H_7_N_2_)_2_PbCl_4_, respectively. g) Pressure‐dependent octahedral‐distortion parameters ($\delta_{oct}^{2}$) in (C_7_H_7_N_2_)_2_PbBr_4_ and (C_7_H_7_N_2_)_2_PbCl_4_, respectively.

The optical properties of 2D HMHs are known to directly depend on their structural variations. Thus, in situ high‐pressure Raman and angle‐dispersive X‐ray diffraction (ADXRD) experiments are conducted to track the structure evolutions of (C_7_H_7_N_2_)_2_PbBr_4_ and (C_7_H_7_N_2_)_2_PbCl_4_ at different pressures (**Figure** **3**; Figures S10 and S11, Supporting Information). As shown in Figure S10 (Supporting Information), all octahedra‐related Raman peaks of (C_7_H_7_N_2_)_2_PbBr_4_ shift to a higher frequency with increasing pressure, indicating the shortening of bond lengths.^[^ ^19^ ^]^ The high‐pressure Raman spectra reveal that (C_7_H_7_N_2_)_2_PbBr_4_ does not exhibit discernible vibrational mode above 3 GPa (Figure S10, Supporting Information), indicating that the formation of amorphous structure. The optical and Raman results show that the structural change and PL tunability of (C_7_H_7_N_2_)_2_PbBr_4_ are reversible during this mild compression, and become irreversible at higher pressures. In the case of (C_7_H_7_N_2_)_2_PbCl_4_, a new octahedra‐related vibrational mode emerges at 0.5 GPa (marked with red star) and rapidly shifts toward high‐frequency region before 1.4 GPa. This new mode is possibly associated with the structural distortion of inorganic [PbCl_6_]^4−^ (Figure S11a,d, Supporting Information). Furthermore, the Raman peaks of (C_7_H_7_N_2_)_2_PbCl_4_ broaden with reduced intensity beyond 6.0 GPa, indicating a gradual transition to amorphous structure.

In high pressure ADXRD experiments of (C_7_H_7_N_2_)_2_PbBr_4_ and (C_7_H_7_N_2_)_2_PbCl_4_, all the diffraction peaks continuously shift to higher angles, indicating a gradual contraction of the lattice (Figures S12 and S13, Supporting Information). The distinctive shift rates of each diffraction peak lead to the separation and merging of some diffraction peaks (Figure 3a,b). The results of in situ high‐pressure ADXRD show that (C_7_H_7_N_2_)_2_PbBr_4_ and (C_7_H_7_N_2_)_2_PbCl_4_ can maintain the monoclinic phase under ambient condition and high pressure (Figure 3a,b; Figure S14, Supporting Information). However, with increasing pressure to 2.9 GPa, the diffraction peaks of (C_7_H_7_N_2_)_2_PbBr_4_ are gradually broadened, which is also indicative of pressure‐induced amorphization. The continuous change in bond length, bond angle, and cell parameters under pressure further indicate the absence of phase transition in (C_7_H_7_N_2_)_2_PbBr_4_ and (C_7_H_7_N_2_)_2_PbCl_4_ under mild compression (Figures S12, S13 and S15, Supporting Information). The anisotropic contraction of (C_7_H_7_N_2_)_2_PbBr_4_ and (C_7_H_7_N_2_)_2_PbCl_4_ with the increasing pressure can be attributed to a relatively higher susceptibility to compression along the direction perpendicular to the organic layer (Figure S16, Supporting Information). This observation is also consistent with the greater reductions in bond lengths perpendicular to the organic layer in these two HMHs (Figure S15, Supporting Information).

As for (C_7_H_7_N_2_)_2_PbBr_4_ and (C_7_H_7_N_2_)_2_PbCl_4_ with typical 2D hybrid structure, three key structural parameters including the distance between neighboring inorganic layers, the distance between N atom in organic cation and Cl/Br atom in metal–halide anion, as well as the degree of inorganic octahedral distortion, are correspondingly analyzed, to uncover the factors affecting their excitonic emissions (Figure 3c–g). As known, the smaller distance between neighboring inorganic layers indicates increased structural stiffness that inhibits the transient lattice distortion in the excited state under light irradiation, which prevents the occurrence of STE emission in HMHs.^[^ ^20^ ^]^ The other distance between the N atom and the halogen atom determines the interaction force between the organic ligand and inorganic octahedron, commonly known as the Coulomb force. A larger Coulomb force (smaller interatomic distance) can induce exciton self trapping at the room temperature, resulting in an efficient broadband STE emission.^[^ ^21^ ^]^ On the other hand, increased octahedral distortions in 2D HMHs have been also reported to promote exciton self‐trapping, facilitating STE emission.^[^ ^16^ ^]^ Compared with (C_7_H_7_N_2_)_2_PbCl_4_ at 1 atm, (C_7_H_7_N_2_)_2_PbBr_4_ exhibits a smaller interlayer spacing, a larger distance between N atom and halogen atom (smaller Coulomb force), and a smaller octahedral distortion (Figure 3e–g). Altogether, (C_7_H_7_N_2_)_2_PbBr_4_ possesses a low electron–phonon coupling strength, which is not favorable to the formation of STE states. Thus, only FE emission is observed in (C_7_H_7_N_2_)_2_PbBr_4_. In contrast, (C_7_H_7_N_2_)_2_PbCl_4_ exhibits both FE emission and STE emission at ambient conditions, due to a much higher electron–phonon coupling strength.

Under compression, the interlayer spacing and the octahedral distortion of (C_7_H_7_N_2_)_2_PbBr_4_ are consistently smaller than those of (C_7_H_7_N_2_)_2_PbCl_4_ (Figure 3e,g). The distance between N atom and Br atom in (C_7_H_7_N_2_)_2_PbBr_4_ is consistently larger than that between N atom and Cl atom in (C_7_H_7_N_2_)_2_PbCl_4_ (Figure 3f), which results in a weaker Coulomb force in (C_7_H_7_N_2_)_2_PbBr_4_. Consequently, (C_7_H_7_N_2_)_2_PbBr_4_ still only exhibits FE emission under high pressure, due to its consistent greater structural stiffness, smaller octahedral distortion and smaller Coulomb force than those in (C_7_H_7_N_2_)_2_PbCl_4_. Based on the analyses of these three paratemeters of (C_7_H_7_N_2_)_2_PbCl_4_ (Figure 3e–g), the structural stiffness, Coulomb force, and inorganic octahedral distortion ($\delta_{oct}^{2}$) of (C_7_H_7_N_2_)_2_PbCl_4_ increase simultaneously as the pressure increase to 1.6 GPa. During this process, STE emission gradually diminishes while FE emission gradually enhances, indicating the dominant role of increased structural stiffness in facilitating FE emission under initial compression. Upon further compression to 5.7 GPa, the octahedral distortion ($\delta_{oct}^{2}$) changes slightly, while the structural stiffness and Coulomb force continue to increase, resulting in the intensity variations of FE emission. At the critical pressure of 6.0 GPa, the octahedral distortion parameter ($\delta_{oct}^{2}$) of (C_7_H_7_N_2_)_2_PbCl_4_ begins to increase obviously, which corresponds well with the emergence of STE‐2 emission. These observations indicate that the increased octahedral distortion and Coulomb force under higher pressure are conducive to exciton self‐trapping to generate STE‐2 emission in (C_7_H_7_N_2_)_2_PbCl_4_. It should be noted that the emissions of STE‐1 and STE‐2 are different. At ambient pressure, (C_7_H_7_N_2_)_2_PbCl_4_ exhibits a stronger electron–phonon coupling strength, which leads to the emergence of STE‐1 emission. Although the reduction of layer spacing results in a stiffer structure under high pressure, the degree of octahedral distortions and the organic cation‐Cl interaction increase, finally leading to the emergence of STE‐2 emission.

**Figure 4:**
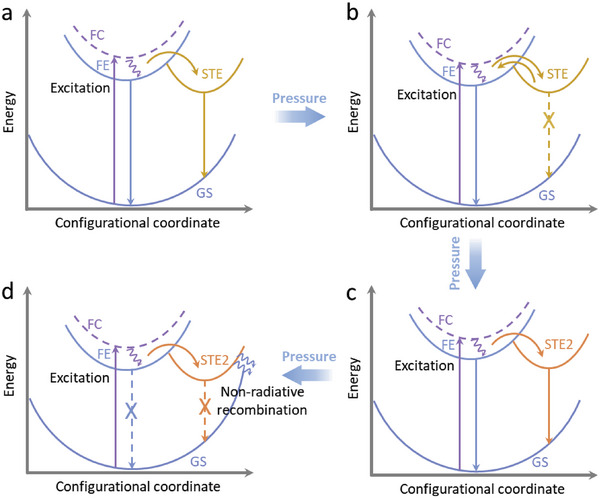
Schematic illustration of pressure‐dependent transitions between STE emission and FE emission in (C_7_H_7_N_2_)_2_PbCl_4_. Free carrier state (FC), GS, free‐exciton state (FE), and STE state.

Based on above analyses, the mechanisms of different excitonic emission modes in (C_7_H_7_N_2_)_2_PbCl_4_ under high pressure are illustrated in **Figure** **4**. At ambient conditions, the photon absorption at 355 nm excites electrons from ground state (GS) to excited state, and strong electron–phonon coupling leads to transient deformation of the inorganic lead halide octahedra, generating STE emission (Figure 4a). Upon compression to 1.6 GPa, the interlayer spacing of inorganic octahedra rapidly decreases, leading to a highly stiff structure. The increased stiffness reduces the strength of electron–phonon coupling in HMHs, inhibiting the electron relaxation to the self‐trapped state and facilitating the electron escape from the self‐trapped state (Figure 4b). The Huang–Rhys factor (*S*) is commonly used to quantify the strength of electron–phonon coupling in HMHs^[^ ^17^, ^22^ ^]^ (see the Supporting Information). As the pressure increases to 2.0 GPa, the

*S* factor calculated based on the STE emission in (C_7_H_7_N_2_)_2_PbCl_4_ decreases from 181 at 1 atm^[^ ^15^ ^]^ to 138.7 at 2.0 GPa (Figure S18c,d, Supporting Information), indicating a decrease in the strength of electron–phonon coupling. This variation is further supported by the decrease of the energy difference (Δλ) between STE emission and FE emission (Figure S19, Supporting Information) in the pressure range.^[^ ^23^ ^]^ With the increasing pressure to 3.3 GPa, FE emission intensity in (C_7_H_7_N_2_)_2_PbCl_4_ continues to increase, possibly due to the inhibition of non‐radiative/trap recombination (Figure S4a, Supporting Information).^[^ ^9a^ ^]^ As discussed above, further compression to 6.0 GPa results in an increase in the octahedral parameter ($\delta_{oct}^{2}$) and the Coulomb force, leading to the emergence of STE‐2 emission (Figure 4c). Additionally, the binding energy calculated based on the STE emission in (C_7_H_7_N_2_)_2_PbCl_4_ increases from 47.1 meV at 3.4 GPa to 83.9 meV at 7.0 GPa (Figure S20c,d, Supporting Information). The higher STE exciton binding energy indicates self‐trapping exciton is more energetically stable, which could also explain the emergence of the STE‐2 emission. As the pressure exceeds a certain threshold, the crystallinity of (C_7_H_7_N_2_)_2_PbCl_4_ begins to decrease, leading to an increase in non‐radiative recombinations and eventually resulting in PL quenching (Figure 4d). In brief, the increased structural stiffness inhibits the transient lattice distortion in excited states, while the increased octahedral distortion in ground states and the heightened Coulomb force can induce self‐trapping of excitons. Rather than relying on a single factor to determine excitonic emission in 2D HMHs, it is crucial to consider whether these variables cooperate or compete.

## 3 Conclusion 

In summary, the excitonic emissions of two structurally similar HMHs, (C_7_H_7_N_2_)_2_PbBr_4_ and (C_7_H_7_N_2_)_2_PbCl_4_, are regulated by high pressure. The intrinsic structure‐property relationship is established, and the factors influencing the types of excitonic emissions are discussed accordingly. Under lower pressure, the dominance of structural stiffness results in a transition from STE emissions to FE emissions for (C_7_H_7_N_2_)_2_PbCl_4_. Under higher pressure, the increased octahedral distortion and heightened Coulomb force between organic cations and inorganic octahedra induce exciton self‐trapping, leading to the development of STE‐2 emission in (C_7_H_7_N_2_)_2_PbCl_4_. Additionally, the replacement of chlorine with bromine in (C_7_H_7_N_2_)_2_PbBr_4_ weakens the Coulomb interaction between the organic cation and inorganic octahedra and increases structural stiffness, both of which results in FE emissions under ambient condition and high pressure. This study highlights the importance of considering various aspects when analyzing excitonic emission behavior and provides insightful information on excitonic emission in 2D Pb‐based HMHs.

## Conflict of Interest

The authors declare no conflict of interest.

## Supporting information

Supporting Information

## Data Availability

Research data are not shared.

## References

[1] a) M. D. Smith , B. A. Connor , H. I. Karunadasa , Chem. Rev. 2019, 119, 3104; 30689364 10.1021/acs.chemrev.8b00477

[2] K. Leng , I. Abdelwahab , I. Verzhbitskiy , M. Telychko , L. Chu , W. Fu , X. Chi , N. Guo , Z. Chen , Z. Chen , C. Zhang , Q.‐H. Xu , J. Lu , M. Chhowalla , G. Eda , K. P. Loh , Nat. Mater. 2018, 17, 908. 30202109 10.1038/s41563-018-0164-8

[3] a) J. Tan , D. Li , J. Zhu , N. Han , Y. Gong , Y. Zhang , Nanoscale 2022, 14, 16394; 36317508 10.1039/d2nr03935d

[4] a) C. Pareja‐Rivera , J. A. Morán‐Muñoz , A. P. Gómora‐Figueroa , V. Jancik , B. Vargas , J. Rodríguez‐Hernández , D. Solis‐Ibarra , Chem. Mater. 2022, 34, 9344;

[5] a) B. Dhanabalan , G. Biffi , A. Moliterni , V. Olieric , C. Giannini , G. Saleh , L. Ponet , M. Prato , M. Imran , L. Manna , R. Krahne , S. Artyukhin , M. P. Arciniegas , Adv. Mater. 2021, 33, 2008004; 10.1002/adma.202008004 PMC11468748 33644923

[6] J. Yu , J. Kong , W. Hao , X. Guo , H. He , W. R. Leow , Z. Liu , P. Cai , G. Qian , S. Li , X. Chen , X. Chen , Adv. Mater. 2019, 31, 1806385. 10.1002/adma.201806385 30556251

[7] a) S. Jiang , Y. Luan , J. I. Jang , T. Baikie , X. Huang , R. Li , F. O. Saouma , Z. Wang , T. J. White , J. Fang , J. Am. Chem. Soc. 2018, 140, 13952; 30265811 10.1021/jacs.8b09316

[8] Z. Ma , Z. Liu , S. Lu , L. Wang , X. Feng , D. Yang , K. Wang , G. Xiao , L. Zhang , S. A. T. Redfern , B. Zou , Nat. Commun. 2018, 9, 4506. 30374042 10.1038/s41467-018-06840-8 PMC6206024

[9] a) S. Guo , Y. Zhao , K. Bu , Y. Fu , H. Luo , M. Chen , M. P. Hautzinger , Y. Wang , S. Jin , W. Yang , X. Lü , Angew. Chem., Int. Ed. 2020, 59, 17533; 10.1002/anie.202001635 32627251

[10] a) Z. Ma , F. Li , L. Sui , Y. Shi , R. Fu , K. Yuan , G. Xiao , B. Zou , Adv. Opt. Mater. 2020, 8, 2000713;

[11] M. Chen , S. Guo , K. Bu , S. Lee , H. Luo , Y. Wang , B. Liu , Z. Yan , H. Dong , W. Yang , B. Ma , X. Lü , Matter Radiat. at Extremes 2021, 6, 058401.

[12] Y. Wang , S. Guo , H. Luo , C. Zhou , H. Lin , X. Ma , Q. Hu , M.‐H. Du , B. Ma , W. Yang , X. Lü , J. Am. Chem. Soc. 2020, 142, 16001. 32870668 10.1021/jacs.0c07166

[13] L. Kong , G. Liu , J. Gong , L. Mao , M. Chen , Q. Hu , X. Lü , W. Yang , M. G. Kanatzidis , H. K. Mao , Proc. Natl. Acad. Sci. USA. 2020, 117, 16121. 32601216 10.1073/pnas.2003561117 PMC7368378

[14] L. Zhang , L. Wu , K. Wang , B. Zou , Adv. Sci. 2019, 6, 1801628. 10.1002/advs.201801628 PMC6343061 30693191

[15] Y. Han , J. Yin , G. Cao , Z. Yin , Y. Dong , R. Chen , Y. Zhang , N. Li , S. Jin , O. F. Mohammed , B.‐B. Cui , Q. Chen , ACS Energy Lett. 2022, 7, 453.

[16] B. Febriansyah , T. Borzda , D. Cortecchia , S. Neutzner , G. Folpini , T. M. Koh , Y. Li , N. Mathews , A. Petrozza , J. England , Angew. Chem., Int. Ed. 2020, 59, 10791. 10.1002/anie.201915708 32271981

[17] Q. Li , B. Xu , Z. Chen , J. Han , L. Tan , Z. Luo , P. Shen , Z. Quan , Adv. Funct. Mater. 2021, 3, 2104923.

[18] a) J. Yin , R. Naphade , L. Gutiérrez Arzaluz , J.‐L. Brédas , O. M. Bakr , O. F. Mohammed , ACS Energy Lett. 2020, 5, 2149;

[19] S. Guo , K. Bu , J. Li , Q. Hu , H. Luo , Y. He , Y. Wu , D. Zhang , Y. Zhao , W. Yang , M. G. Kanatzidis , X. Lü , J. Am. Chem. Soc. 2021, 143, 2545. 33465309 10.1021/jacs.0c11730

[20] a) X. Gong , O. Voznyy , A. Jain , W. Liu , R. Sabatini , Z. Piontkowski , G. Walters , G. Bappi , S. Nokhrin , O. Bushuyev , M. Yuan , R. Comin , D. Mccamant , S. O. Kelley , E. H. Sargent , Nat. Mater. 2018, 17, 550; 29760510 10.1038/s41563-018-0081-x

[21] M. Zhang , L. Zhao , J. Xie , Q. Zhang , X. Wang , N. Yaqoob , Z. Yin , P. Kaghazchi , S. Zhang , H. Li , C. Zhang , L. Wang , L. Zhang , W. Xu , J. Xing , Nat. Commun. 2021, 12, 4890. 34385451 10.1038/s41467-021-25132-2 PMC8361204

[22] H. Luo , S. Guo , Y. Zhang , K. Bu , H. Lin , Y. Wang , Y. Yin , D. Zhang , S. Jin , W. Zhang , W. Yang , B. Ma , X. Lü , Adv. Sci. 2021, 8, 2100786. 10.1002/advs.202100786 PMC8292847 34021734

[23] R. Fu , W. Zhao , L. Wang , Z. Ma , G. Xiao , B. Zou , Angew. Chem., Int. Ed. 2021, 60, 10082. 10.1002/anie.202015395 33759324

