# Supplementary material for: Distinct Excitonic Emissions in 2D (C7H7N2)2PbX4 (X = Cl, Br) under Compression
Source: Adv Sci (Weinh). 2023 Nov 20;11(4):2305597. doi: 10.1002/advs.202305597 (PMC10811510; doi:10.1002/advs.202305597)
Supplement: Supplementary file 1 — Supporting Information [file ADVS-11-2305597-s001.pdf]

ADVANCED SCIENCE Open Access

Supporting Information

for *Adv. Sci.*, DOI 10.1002/advs.202305597

Distinct Excitonic Emissions in 2D $(C_7H_7N_2)_2PbX_4$ (X = Cl, Br) under Compression

*Hai Zhang, Peijie Zhang, Chenlong Xie, Jiang Han, Bin Xu and Zewei Quan**

Distinct Excitonic Emissions in Two-Dimensional $(C_7H_7N_2)_2PbX_4$ (X = Cl, Br) under Compression

Experimental Details

Materials: Lead monoxide (PbO, 99.99%, Aladdin), Benzimidazole ($C_7H_6N_2$, 98.5%, Macklin), Hydrogen bromide (HBr, 40%, Aladdin) and Hydrochloric acid (HCl, 37%, Dongjiang reagent). All chemical reagents in this work were commercially purchased without further purification.

$(C_7H_7N_2)_2PbBr_4$: At room temperature, solid PbO (1.80 mmol, 0.400 g) and benzimidazole (3.60 mmol, 0.425 g) were combined with 25 mL Hydrogen bromide (HBr, 40%) in a glass bottle. The vial was first heated to 110 °C for 30 min to dissolve the solid and then slowly cooled to room temperature at a rate of -1.5 °C·h^{-1} to afford crystals suitable for measurements.

$(C_7H_7N_2)_2PbCl_4$: At room temperature, solid PbO (1.80 mmol, 0.400 g) and benzimidazole (3.60 mmol, 0.425 g) were combined with 25 mL hydrochloric acid (HCl, 37%) in a glass bottle. The vial was first heated to 110 °C for 30 min to dissolve the solid and then slowly cooled to room temperature at a rate of -1.5 °C·h^{-1} to afford crystals suitable for measurements.

High Pressure Experiments:

PL emission spectra under high pressure were recorded by the NOVA spectrometer with the 355 nm laser excitation. Under the Xenon lamp excitation with the wavelength range of 315 nm ~ 375 nm, the CCD camera on the microscope was used to capture the PL microscopic image at the same exposure time. The power-dependent PL emission was monitored by a power meter of Thorlabs PM100D with a standard photodiode power sensor (S120VC). For the temperature-dependent PL experiments under high pressure, the DAC was placed in the cryogenic vacuum chamber and clamped to the cold-finger of the liquid nitrogen flow cryostat (ST-500 from Janis).

High pressure UV−Vis absorption experiments were carried out on the Ocean Optics QE65000 spectrometer with the transmittance method. The transmission spectrum of silicon oil around the sample is subtracted as the background.

The PL lifetime measurements under high pressure were carried out on a confocal microscope with 375 nm picosecond diode lasers (Pico Quant, Taiko PDL M1) as excitation source. The PL signal selected by the monochromator (Princeton Instrument products, HR300MS) was used to generate the detected photons. The time-resolved PL signal was analyzed by single-photon avalanche diode (Pico Quant, Timeharp 260 PCIe Interface).

High pressure Raman spectra were carried out on iRH 550 spectrometers (Horiba

Instruments). The excitation line is 532 nm laser radiation from the diode pumped solid state (DPSS) laser equipment.

In-situ high-pressure angle dispersive X-ray diffraction (ADXRD) experiments were carried out at beamline 15U1, Shanghai Synchrotron Radiation Facility (SSRF). The wavelength of incident X-ray beam was adjusted to be 0.6199 Å and CeO_2 was applied for calibration. The one-dimensional ADXRD spectra were obtained using the Dioptas software.

Data Analyses: The refinements of ADXRD patterns were performed using the Jana program with LeBail module. The peak profile and asymmetry were fitted through Pseudo-Voigt function and Howard correction, respectively.

The pressure-volume (P-V) data were fitted by the third-order Birch-Murnaghan (B-M) equation of state:[1]

$$P(V) = \frac{3B_0}{2}\left[\left(\frac{V_0}{V}\right)^{\frac{7}{3}} - \left(\frac{V_0}{V}\right)^{\frac{5}{3}}\right] \times \left\{1 + \frac{3}{4}(B_0' - 4)\left[\left(\frac{V_0}{V}\right)^{\frac{2}{3}} - 1\right]\right\}$$

Where V_0 is the zero-pressure volume, B_0 is the bulk modulus, and B_0' is a parameter for the pressure derivative. For the structural comparisons in different pressure range, B_0' is fixed at 4.

The octahedral parameters of λ and δ_{oct}^2 were calculated based on the structural parameters at different pressures, according to the following equations:[2]

$$\lambda = \frac{1}{6}\sum_{n=1}^{6}\left(\frac{d_n - d_0}{d_0}\right)$$

$$\delta_{oct}^2 = \frac{1}{11}\sum_{n=1}^{12}(\theta_n - 90^{\circ})^2$$

In these equations, the parameters of d_n and d_0 are the individual bond distance and average bond distance within the octahedra, respectively. The θ_n represents the octahedral angle. Therefore, λ and δ_{oct}^2 could be used to evaluate the variation and deviation of bond lengths and angles in octahedra.

The Huang-Rhys factor (S) is evaluated based on the data fitting of the full width at half maximum (FWHM) at low temperature, following the equation of[3]

$$FWHM = 2.36\sqrt{S}\hbar\omega\sqrt{coth\frac{\hbar\omega}{2k_BT}}$$

where $\hbar\omega$ is the phonon frequency, T is temperature, and k_B represents the Boltzmann constant.

To extract the exciton binding energy, we investigate the thermal quenching of the excitonic emission fitted using[4]

$$I(T) = \frac{I(0K)}{1 + Aexp(-E_b/kT)}$$

where I and A are constants and Eb is the exciton binding energy.

Table S1. Various distortion parameters at room temperature.

	λ	δ_{oct}^2	D_{in}	D_{out}
$(C_7H_7N_2)_2PbCl_4$	5.9 x 10^{-5}	19.6	166°	180°
$(C_7H_7N_2)_2PbBr_4$	1.1 x 10^{-5}	13.0	168°	180°

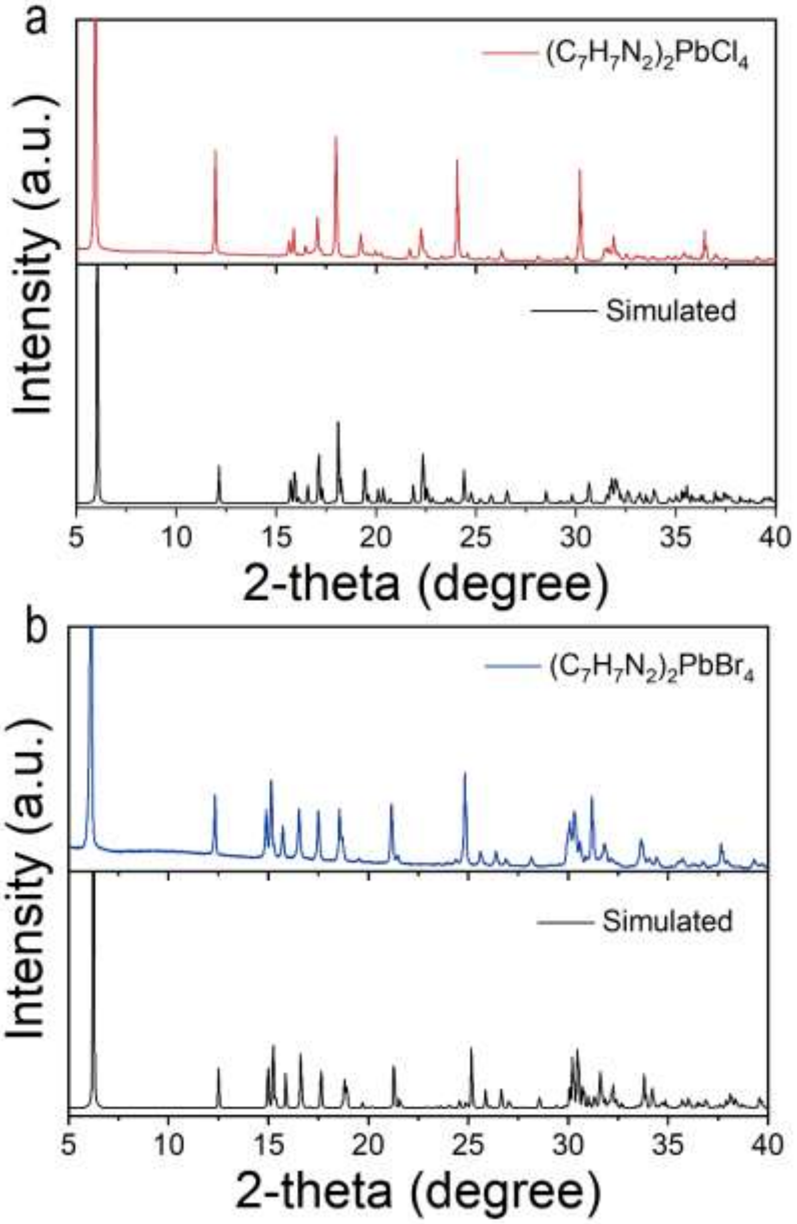

Figure S1. PXRD patterns of $(C_7H_7N_2)_2PbCl_4$ (a), $(C_7H_7N_2)_2PbBr_4$ (b).

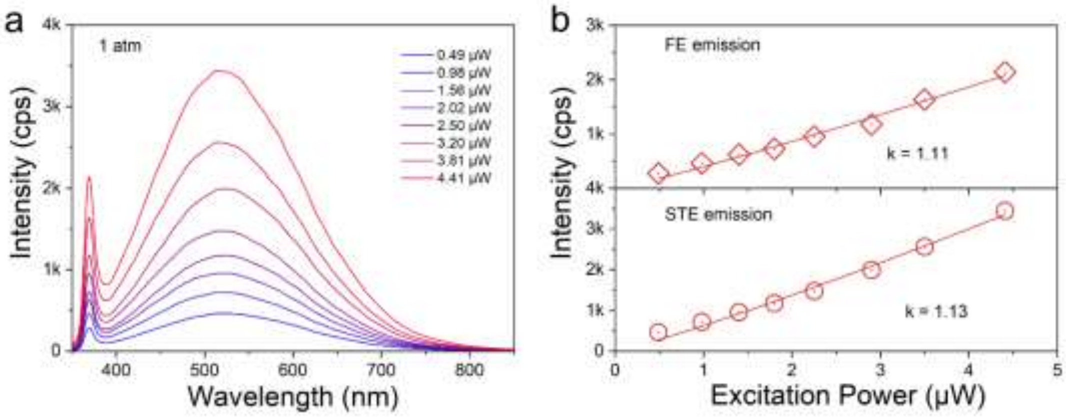

Figure S2. (a) PL intensity of $(C_7H_7N_2)_2PbCl_4$ at 1 atm as a function of power density. (b) The fitted intensity-power data of FE emission and STE emission.

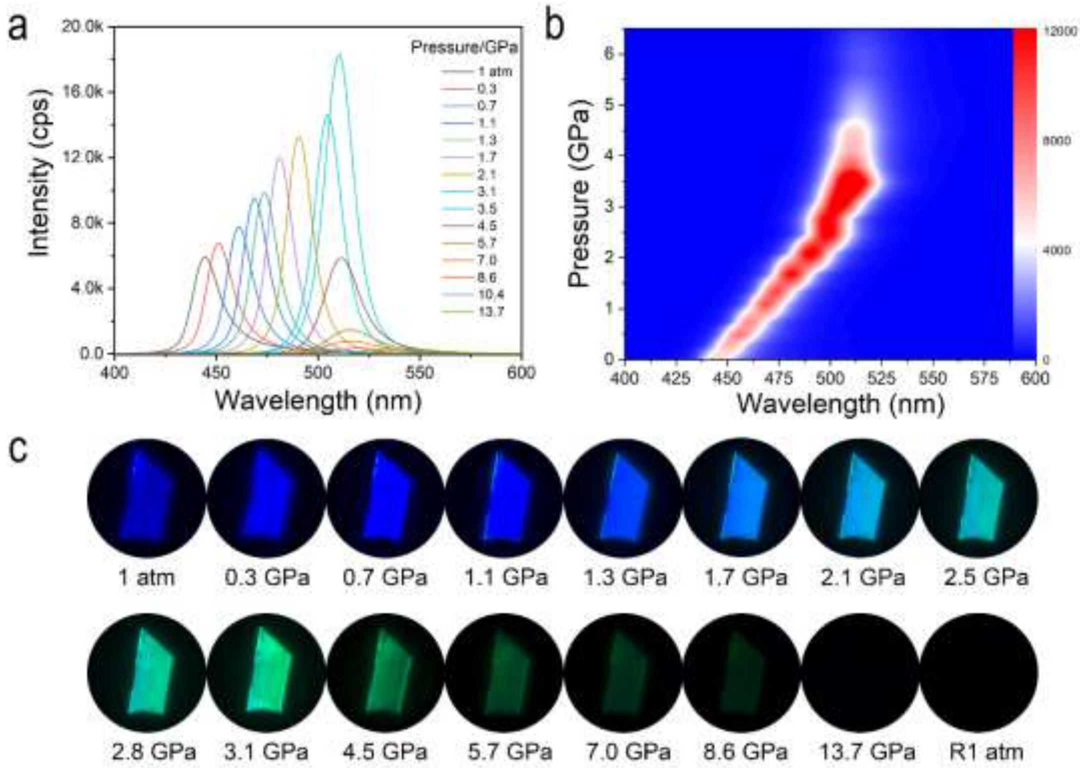

Figure S3. (a) the pressure-dependent PL emission spectra of $(C_7H_7N_2)_2PbBr_4$. The excitation wavelength is 355 nm. (b) The intensity contour maps of the PL emission spectra of $(C_7H_7N_2)_2PbBr_4$. (c) PL emission micrographs of $(C_7H_7N_2)_2PbBr_4$ crystal at different pressures.

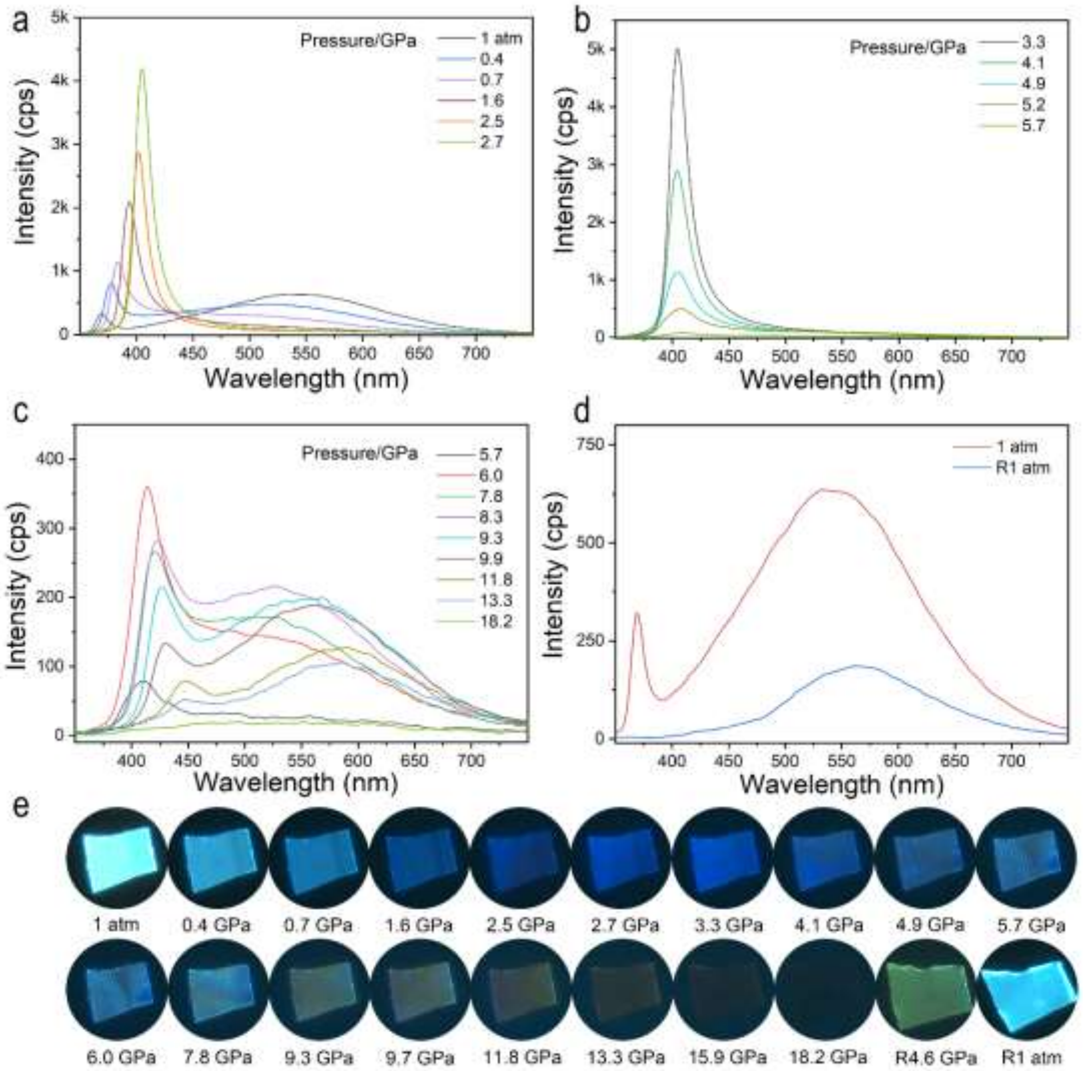

Figure S4. (a)-(c) pressure-dependent PL emission spectra of $(C_7H_7N_2)_2PbCl_4$. The excitation wavelength is 355 nm. (d) Contrast of PL emission spectra between ambient condition and decompression. (e) PL emission micrographs of $(C_7H_7N_2)_2PbCl_4$ at different pressures.

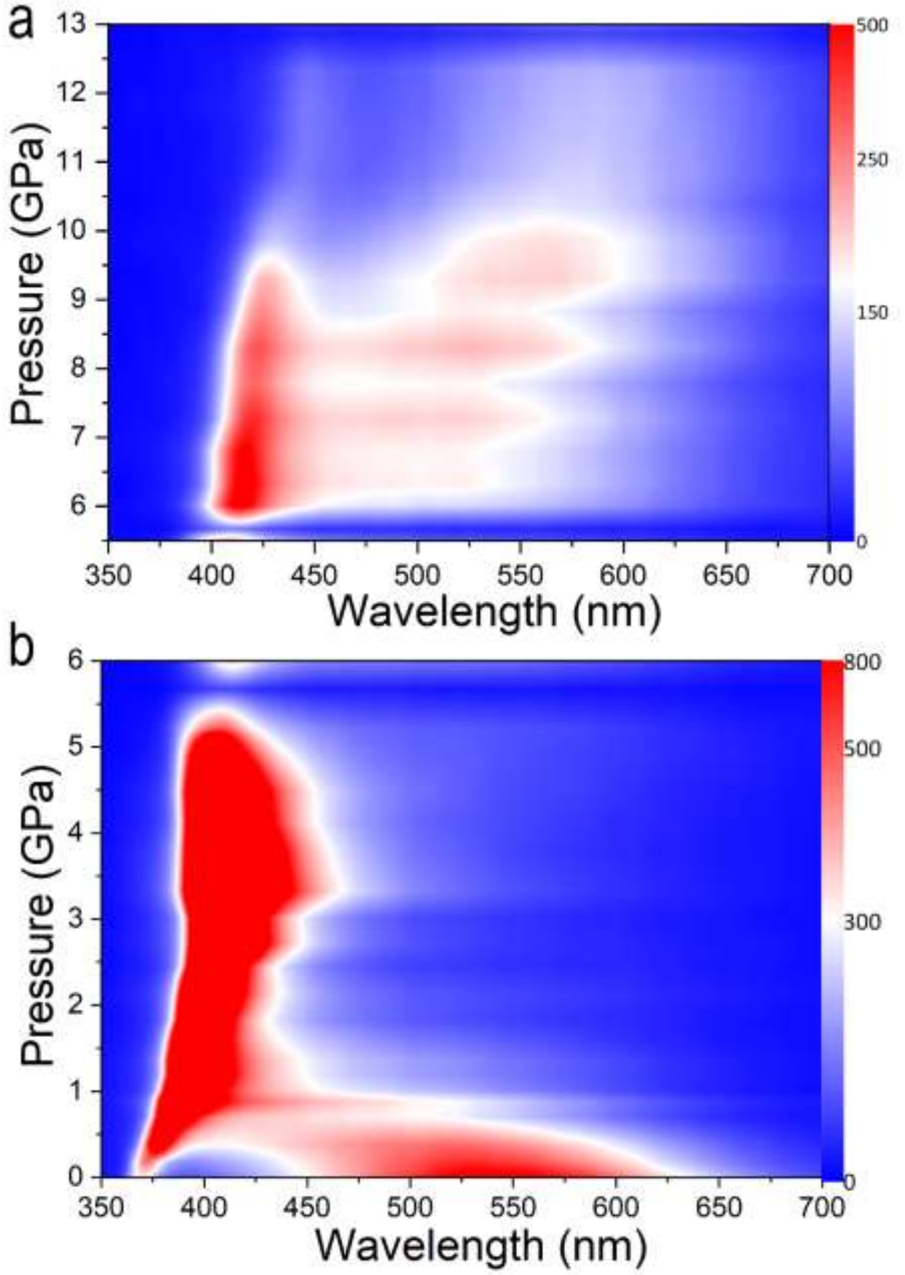

Figure S5. The intensity contour maps of the PL emission spectra of $(C_7H_7N_2)_2PbCl_4$.

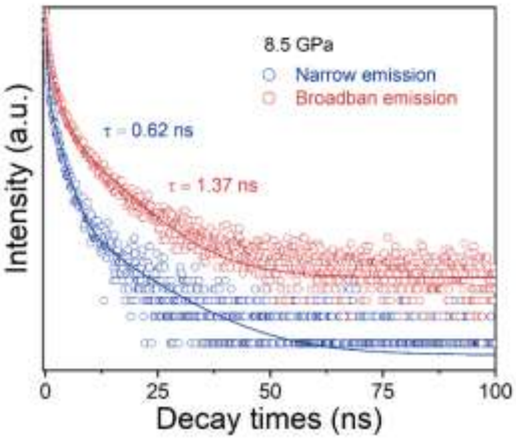

Figure S6. Time-resolved PL decays and fitting curves of $(C_7H_7N_2)_2PbCl_4$ at 8.5 GPa.

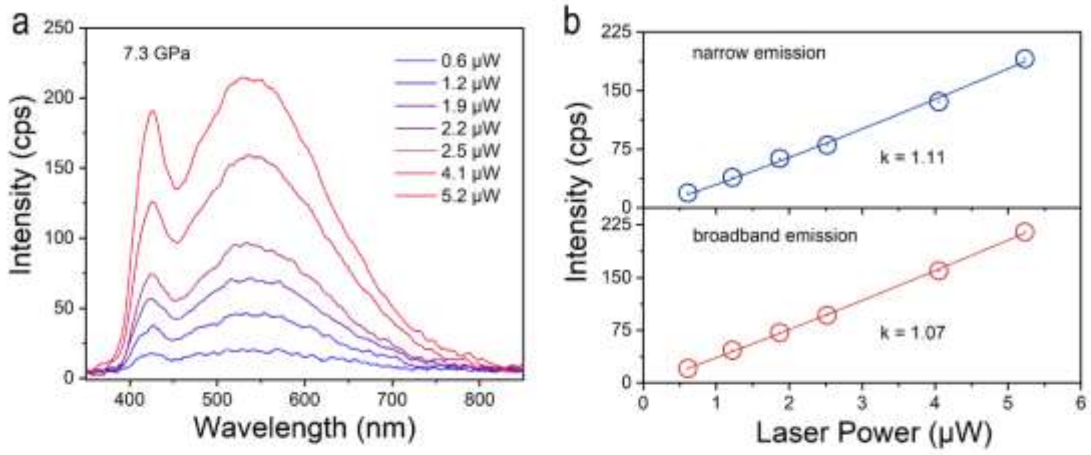

Figure S7. (a) PL intensity of $(C_7H_7N_2)_2PbCl_4$ at 7.3 GPa as a function of power density. (b) The fitted intensity-power data of narrow emission and broadband emission.

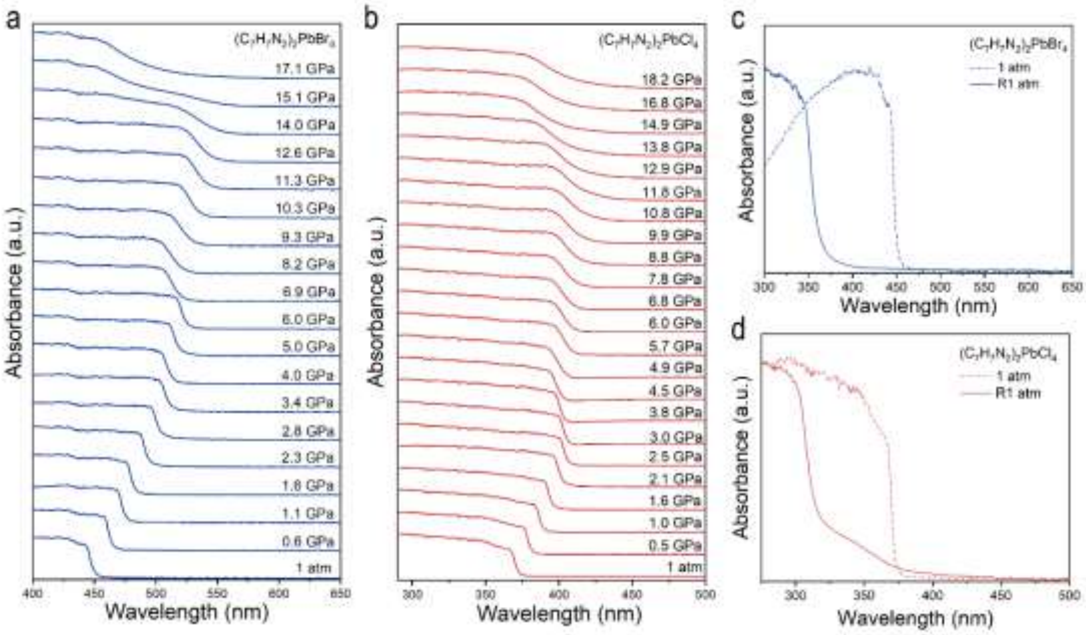

Figure S8. (a) and (b) represent the pressure-dependent absorption spectra of $(C_7H_7N_2)_2PbBr_4$ and $(C_7H_7N_2)_2PbCl_4$, respectively. (c) Comparison of absorption spectra between 1 atm and decompression of $(C_7H_7N_2)_2PbBr_4$. (d) Comparison of absorption spectra between 1 atm and decompression of $(C_7H_7N_2)_2PbCl_4$.

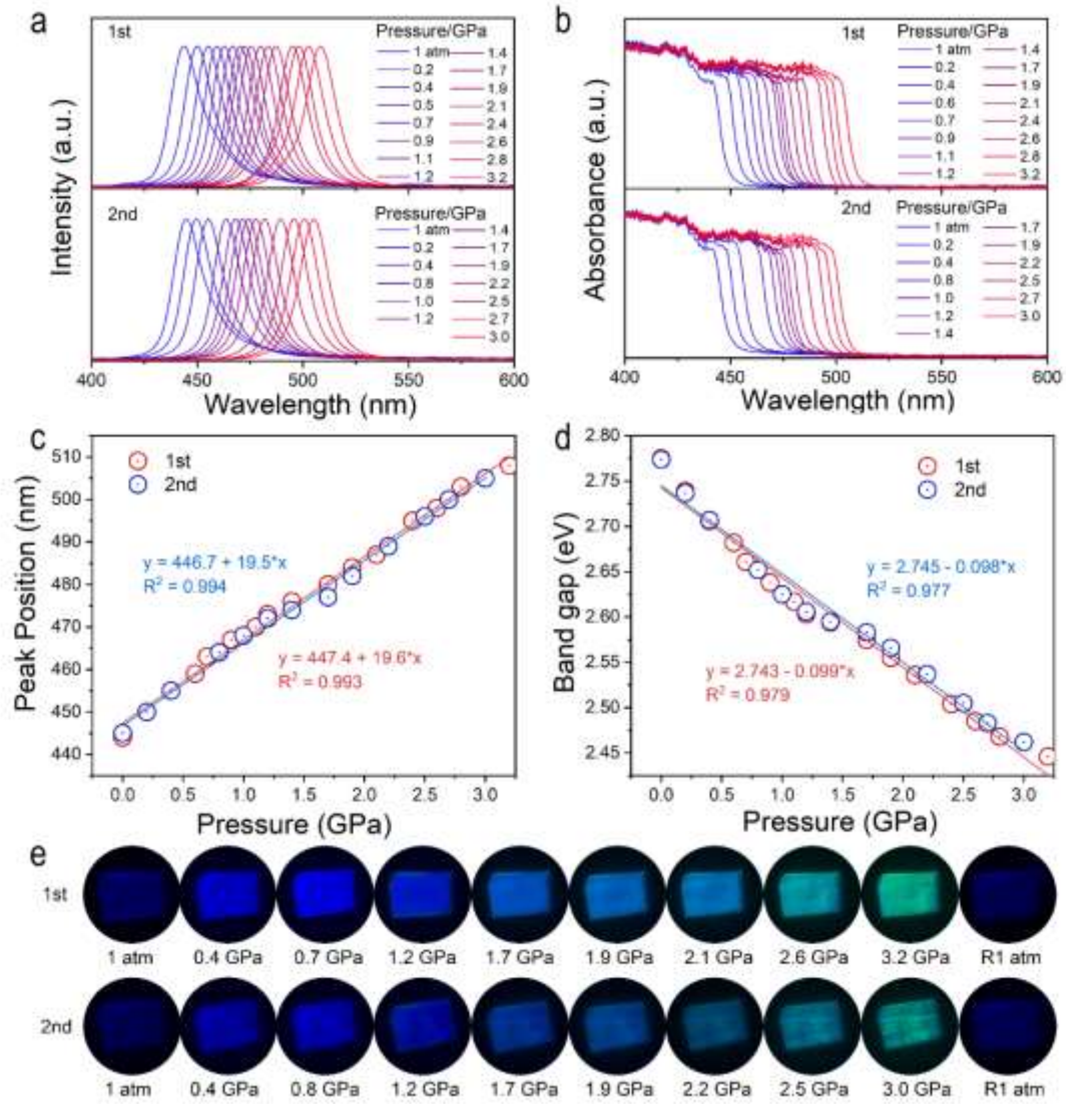

Figure S9. (a) The normalized PL emission intensity of $(C_7H_7N_2)_2PbBr_4$ around 3 GPa. (b) The normalized absorption intensity of $(C_7H_7N_2)_2PbBr_4$ around 3 GPa. (c) and (d) FE peak and band gap position evolution of $(C_7H_7N_2)_2PbBr_4$ around 3 GPa. (e) PL micrographs of $(C_7H_7N_2)_2PbBr_4$ crystal at different pressures.

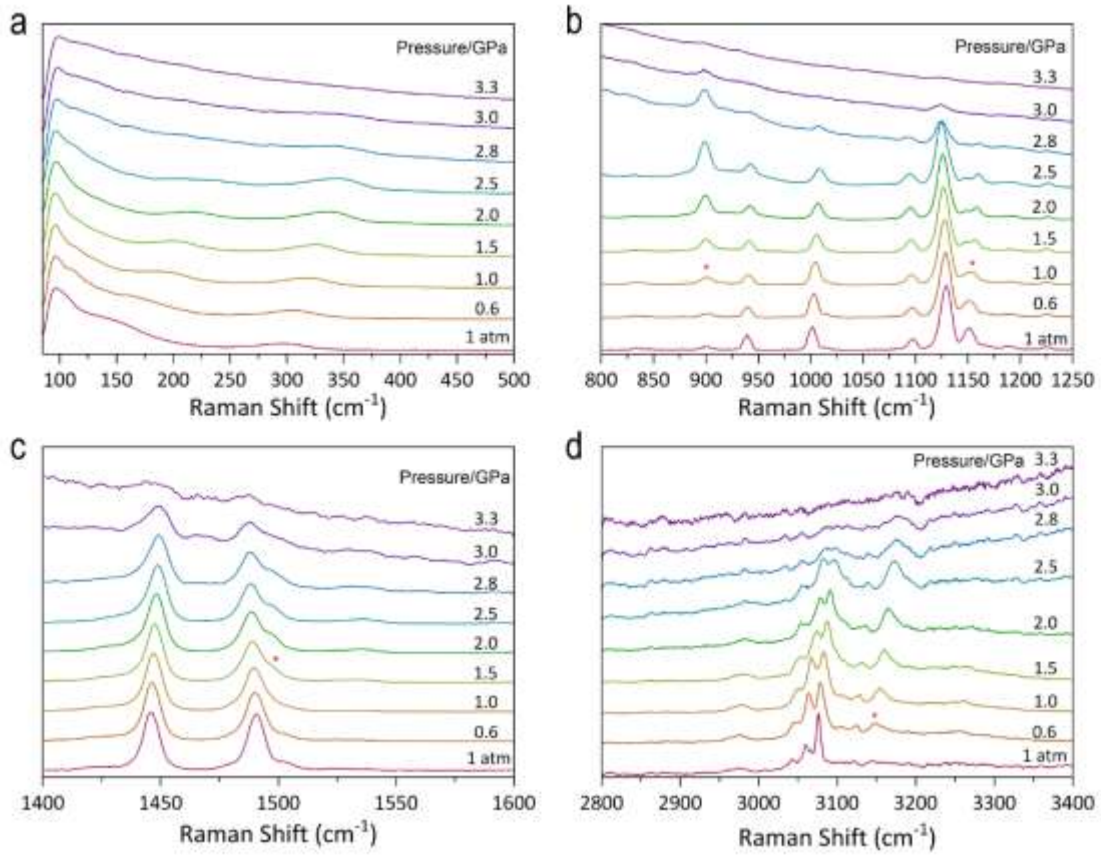

Figure S10. The high-pressure Raman spectra of $(C_7H_7N_2)_2PbBr_4$ between the Raman shift from 80 cm^{-1} to 300 cm^{-1} (a), from 900 cm^{-1} to 1250 cm^{-1}(b), from 1400 cm^{-1} to 1700 cm^{-1}(c), from 2800 cm^{-1} to 3400 cm^{-1}(d).

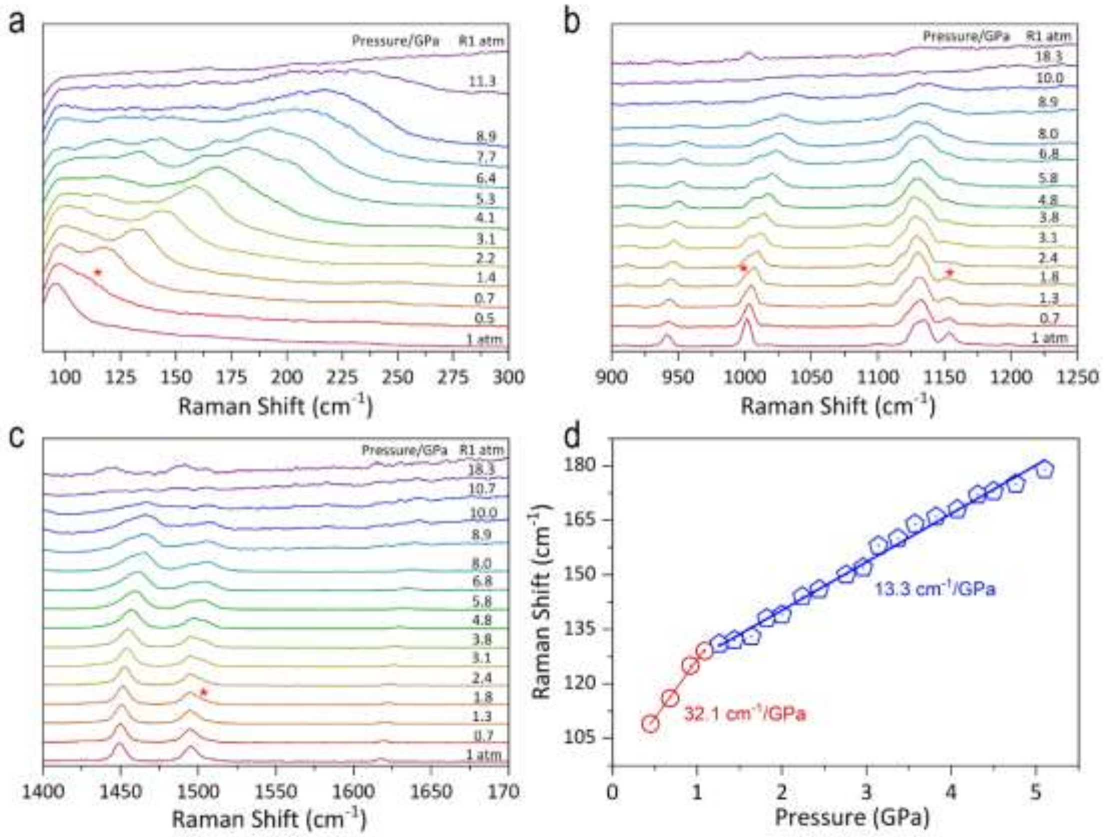

Figure S11. The high-pressure Raman spectra of $(C_7H_7N_2)_2PbCl_4$ between the Raman shift from 80 cm^{-1} to 300 cm^{-1} (a), from 900 cm^{-1} to 1250 cm^{-1} (b), from 1400 cm^{-1} to 1700 cm^{-1} (c). (d) Pressure-dependent Raman shifts of selected vibrations of $(C_7H_7N_2)_2PbCl_4$.

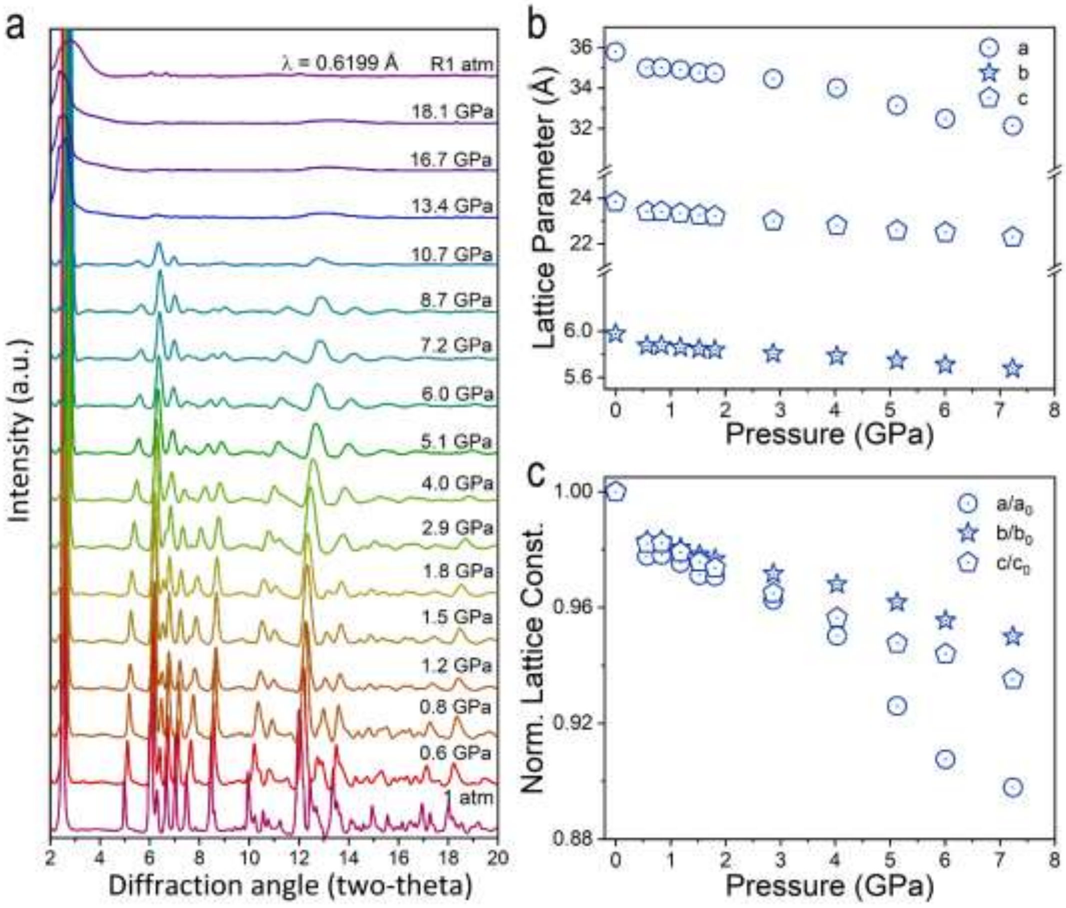

Figure S12. (a) Representative *in-situ* high-pressure ADXRD patterns of $(C_7H_7N_2)_2PbBr_4$. (b) Lattice parameters of $(C_7H_7N_2)_2PbBr_4$ under high pressure. (c) Compressibility along different lattice axes.

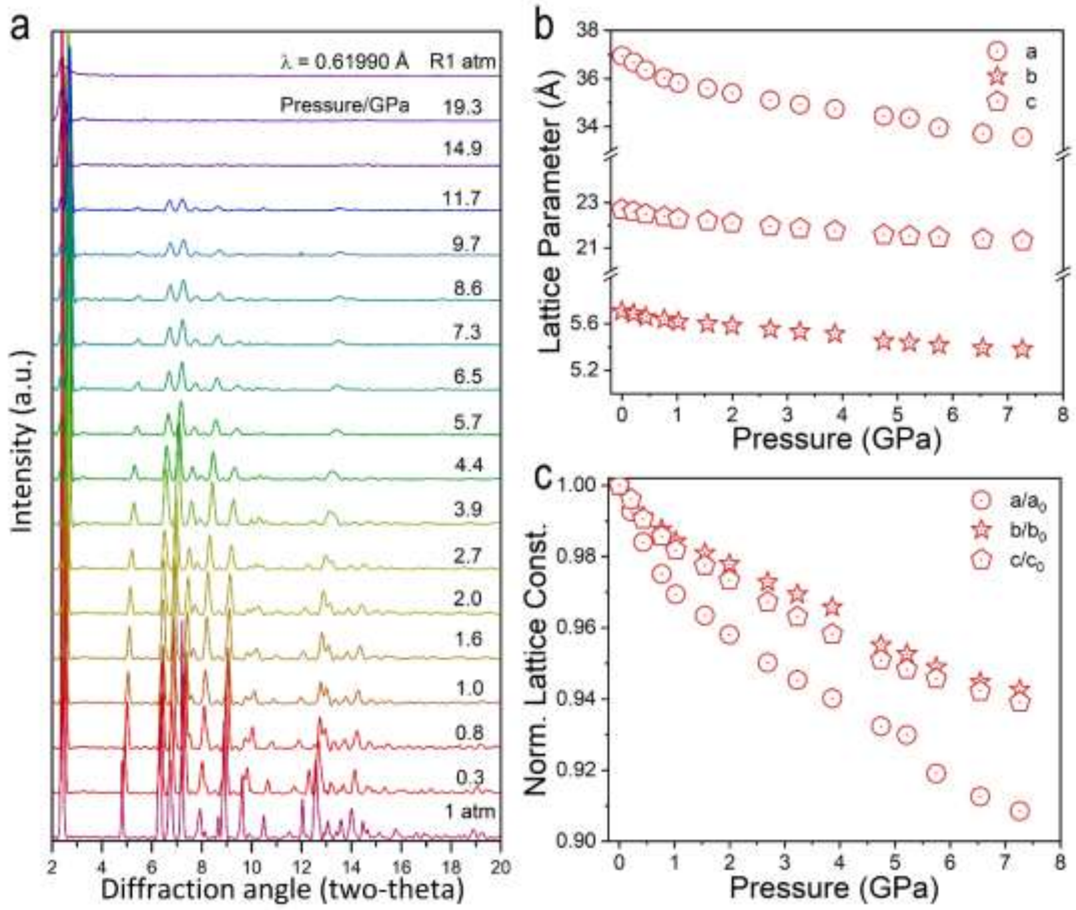

Figure S13. (a) Representative *in-situ* high-pressure ADXRD patterns of $(C_7H_7N_2)_2PbCl_4$. (b) Lattice parameters of $(C_7H_7N_2)_2PbCl_4$ under high pressure. (c) Compressibility along different lattice axes.

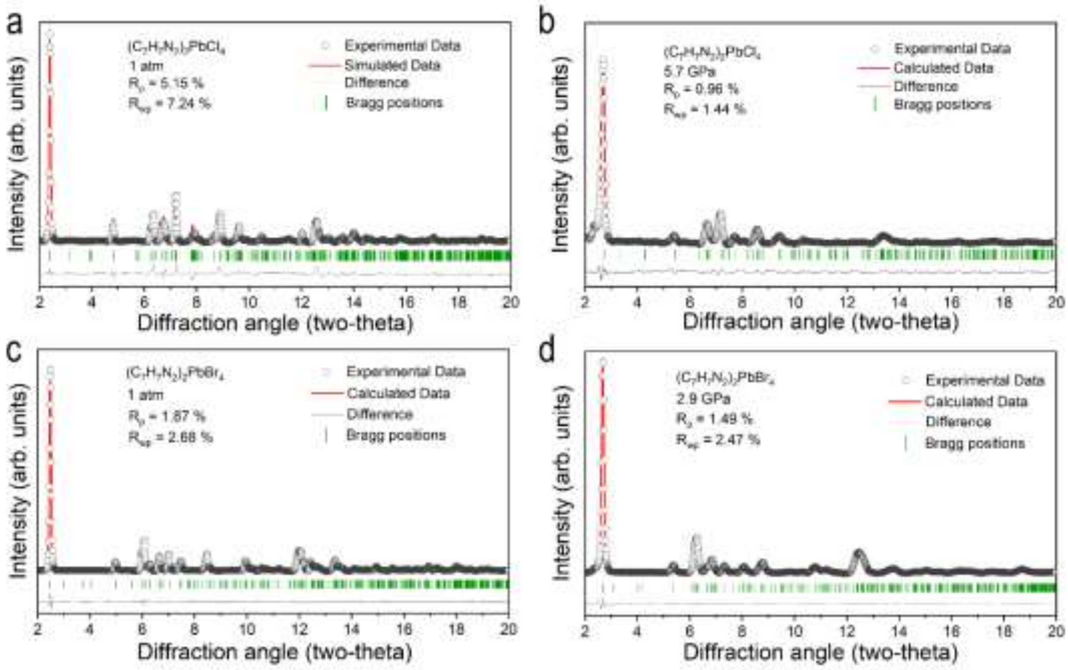

Figure S14. (a) and (b) Refinement results of $(C_7H_7N_2)_2PbCl_4$ at 1 atm and 5.7 GPa. (c) and (d) Refinement results of $(C_7H_7N_2)_2PbBr_4$ at 1 atm and 2.9 GPa.

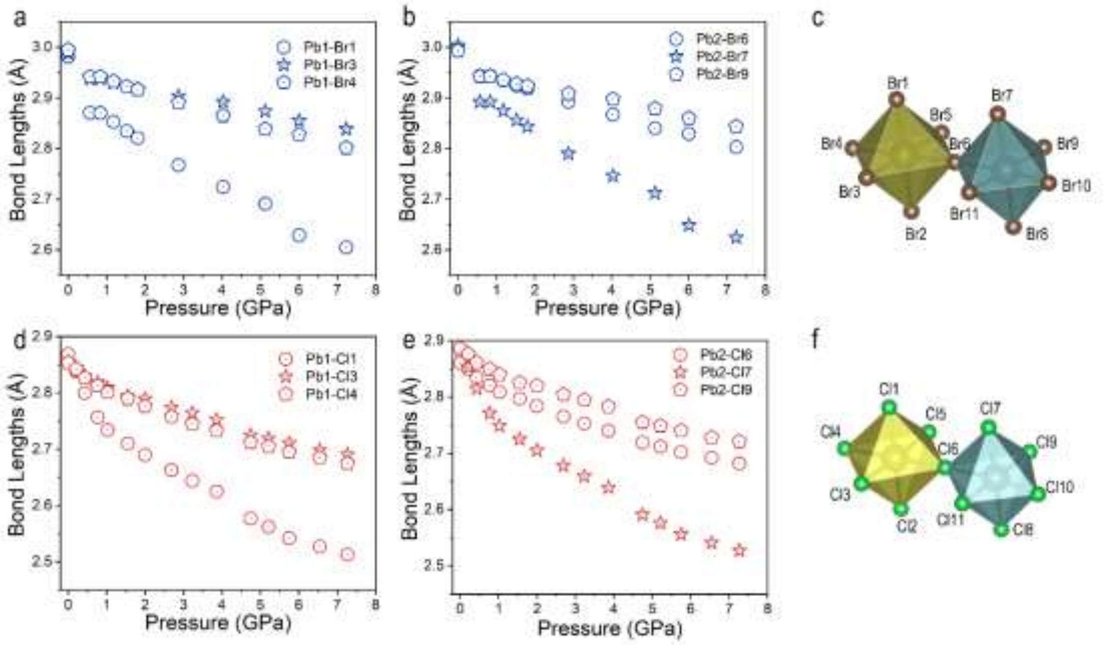

Figure S15. (a) and (b) High-pressure evolution of bond lengths in $(C_7H_7N_2)_2PbBr_4$ octahedra. (d) and (e) High-pressure evolution of bond lengths in $(C_7H_7N_2)_2PbCl_4$ octahedra. (c) and (f) Schematic illustrations of $(C_7H_7N_2)_2PbBr_4$ and $(C_7H_7N_2)_2PbCl_4$ octahedra.

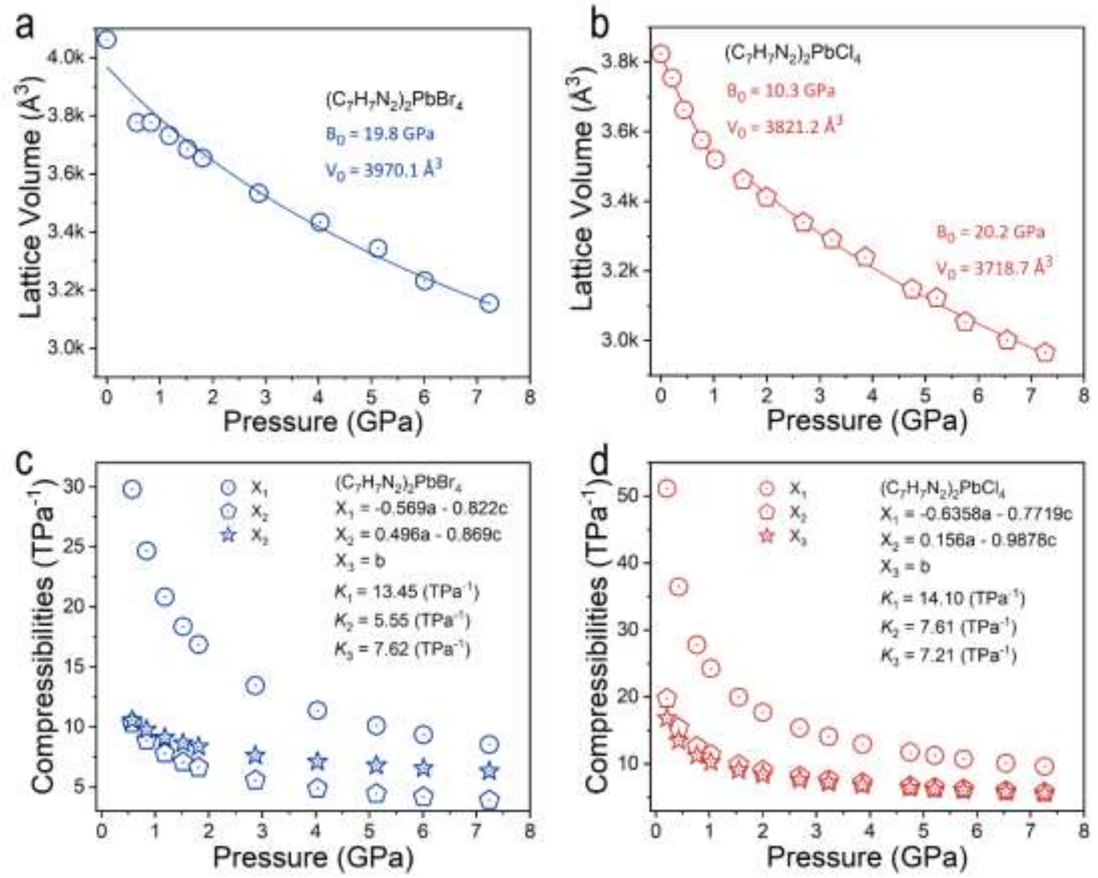

Figure S16. (a) and (b) represent the pressure-dependent lattice-volume evolution of $(C_7H_7N_2)_2PbBr_4$ and $(C_7H_7N_2)_2PbCl_4$ respectively. (c) and (d) High pressure evolution of structural compressibilities of $(C_7H_7N_2)_2PbBr_4$ and $(C_7H_7N_2)_2PbCl_4$, respectively.

B_0 denotes bulk modulus that describes the compressibility of a material under hydrostatic pressure. As shown in Figure S16a, the B_0 of $(C_7H_7N_2)_2PbBr_4$ is determiend to be 19.8 GPa. In contrast, the B_0 of $(C_7H_7N_2)_2PbCl_4$ is 10.3 GPa bellow 1 GPa and 20.2 GPa above 1 GPa (Figure S16b). This difference suggests $(C_7H_7N_2)_2PbCl_4$ has a higher compressibility bellow 1 GPa and a similar compresibility above 1 GPa, compared with $(C_7H_7N_2)_2PbBr_4$.

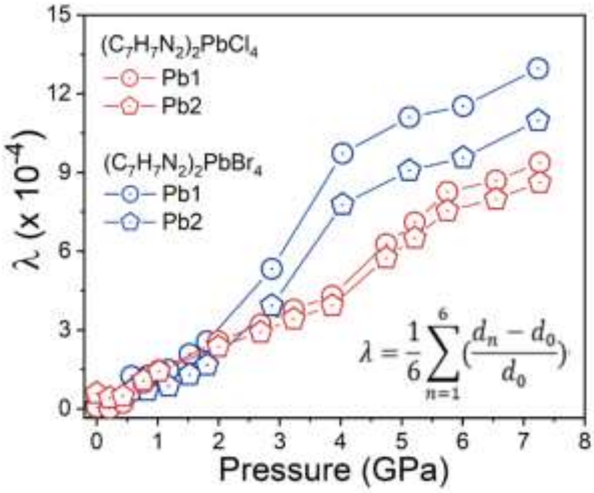

Figure S17. High-pressure octahedral parameters λ of $(C_7H_7N_2)_2PbCl_4$ and $(C_7H_7N_2)_2PbBr_4$, which is calculated based on the equations (insets).

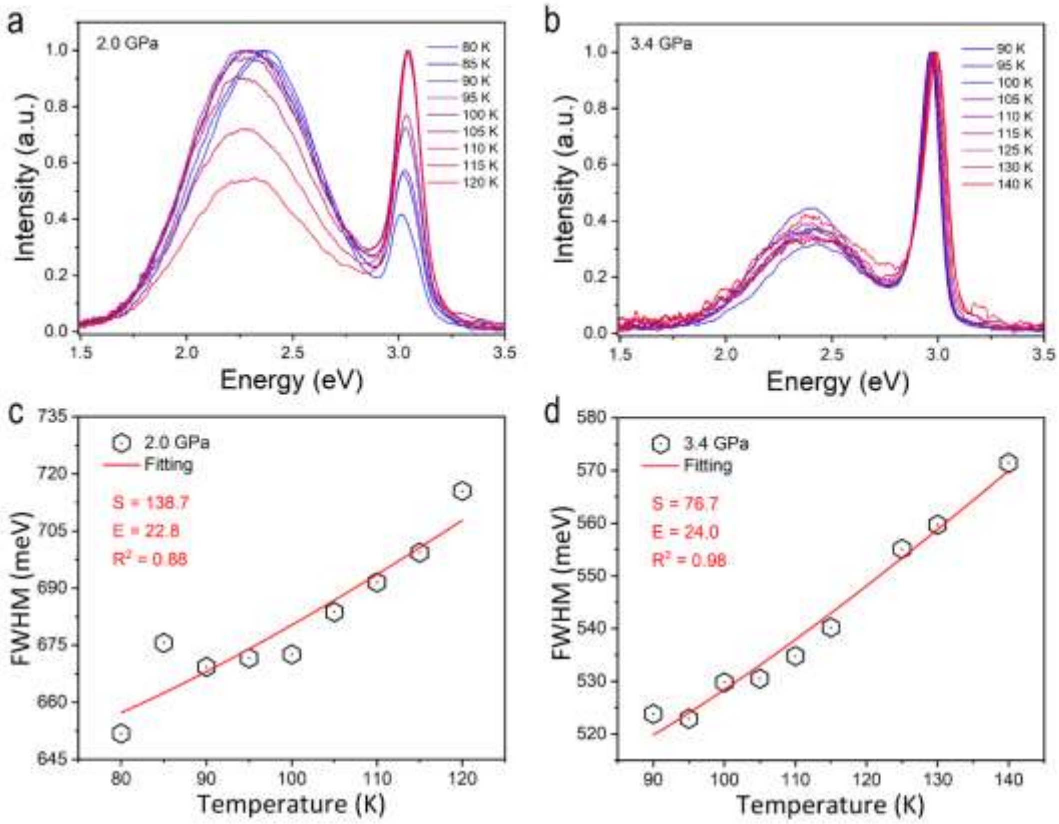

Figure S18. Temperature dependent full width at half maximum (FWHM) of STE emission of $(C_7H_7N_2)_2PbCl_4$ at 2.0 GPa (a) and 3.4 GPa (b), respectively. The Huang-Rhys factor of $(C_7H_7N_2)_2PbCl_4$ measured at at 2.0 GPa (a) and 3.4 GPa (b), respectively. The red lines represent the data fitting of Huang–Rhys factor (S).

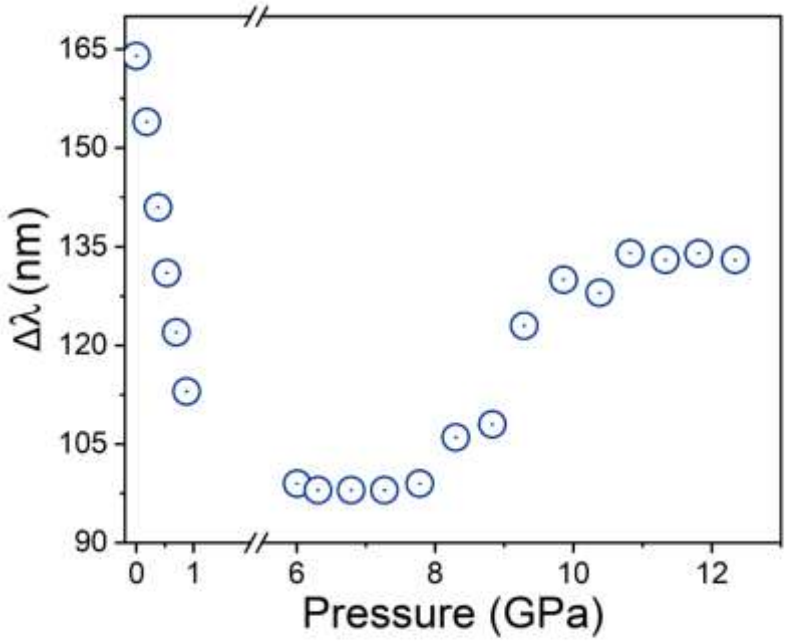

Figure S19. The energy difference (**Δ**λ) between STE emission and FE emission in $(C_7H_7N_2)_2PbCl_4$.

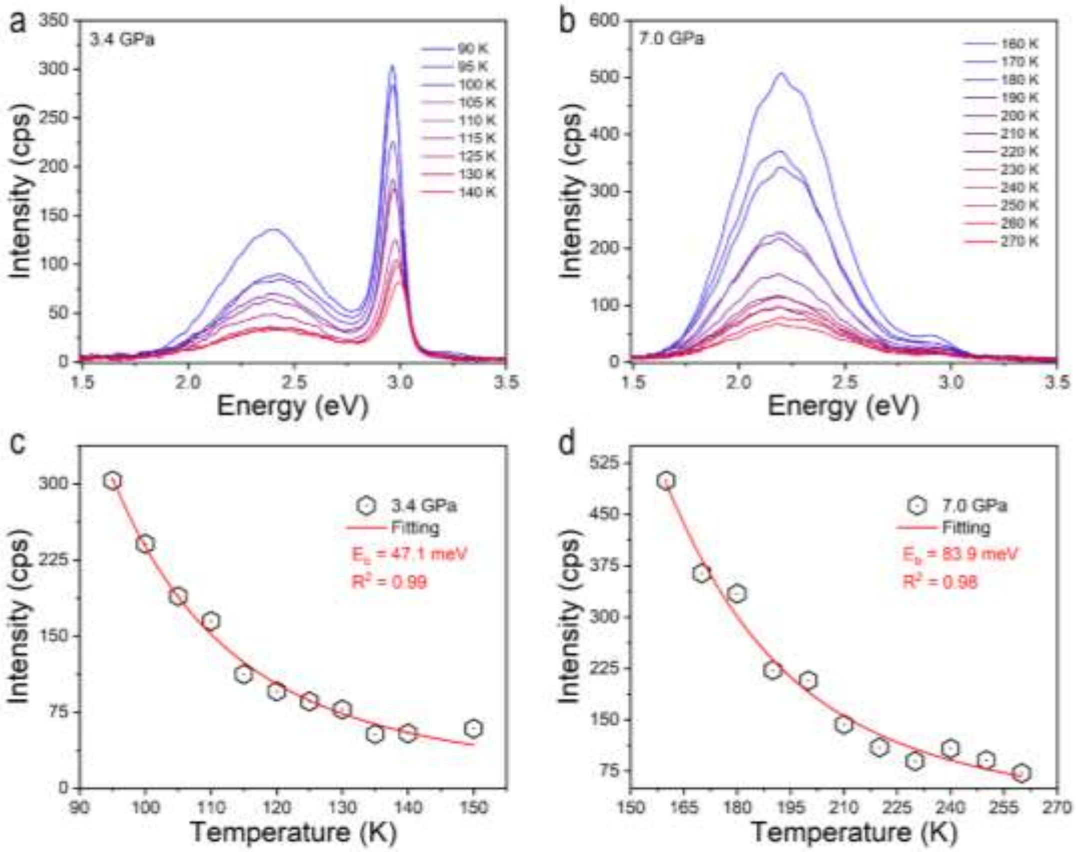

Figure S20. Temperature-dependent intensity of STE emission of $(C_7H_7N_2)_2PbCl_4$ at 3.4 GPa (a) and 7.0 GPa (b), respectively. The corresponding binding energy of self-trapped excitons of $(C_7H_7N_2)_2PbCl_4$ at 3.4 GPa (a) and 7.0 GPa (b), respectively. The red lines represent the data fitting of Exciton binding energy (E_b).

1. Q. Li; B. Xu; Z. Chen; J. Han; L. Tan; Z. Luo; P. Shen; Z. Quan, Excitation-Dependent Emission Color Tuning of 0D $Cs_2InBr_5 \cdot H_2O$ at High Pressure. *Adv. Funct. Mater.* **2021**, 2104923.
2. B. Febriansyah; T. Borzda; D. Cortecchia; S. Neutzner; G. Folpini; T. M. Koh; Y. Li; N. Mathews; A. Petrozza; J. England, Metal Coordination Sphere Deformation Induced Highly Stokes-Shifted, Ultra Broadband Emission in 2D Hybrid Lead-Bromide Perovskites and Investigation of Its Origin. *Angew. Chem. Int. Ed.* **2020**, *59*, 10791-10796.
3. (a)S. Li; J. Luo; J. Liu; J. Tang, Self-Trapped Excitons in All-Inorganic Halide Perovskites: Fundamentals, Status, and Potential Applications. *J. Phys. Chem. Lett.* **2019**, *10*, 1999-2007; (b)J. Luo; X. Wang; S. Li; J. Liu; Y. Guo; G. Niu; L. Yao; Y. Fu; L. Gao; Q. Dong; C. Zhao; M. Leng; F. Ma; W. Liang; L. Wang; S. Jin; J. Han; L. Zhang; J. Etheridge; J. Wang; Y. Yan; E. H. Sargent; J. Tang, Efficient and Stable Emission of Warm-White Light from Lead-Free Halide Double Perovskites. *Nature* **2018**, *563*, 541-545.
4. (a)J.-P. Correa-Baena; L. Nienhaus; R. C. Kurchin; S. S. Shin; S. Wieghold; N. T. Putri Hartono; M. Layurova; N. D. Klein; J. R. Poindexter; A. Polizzotti; S. Sun; M. G. Bawendi; T. Buonassisi, A-Site Cation in Inorganic $A_3Sb_2I_9$ Perovskite Influences Structural Dimensionality, Exciton Binding Energy, and Solar Cell Performance. *Chem. Mater.* **2018**, *30*, 3734-3742; (b)K. Wu; A. Bera; C. Ma; Y. Du; Y. Yang; L. Li; T. Wu, Temperature-Dependent Excitonic Photoluminescence of Hybrid Organometal Halide Perovskite Films. *Phys. Chem. Chem. Phys.* **2014**, *16*, 22476-81.
